# Mouse *Obox* and *Crxos* modulate preimplantation transcriptional profiles revealing similarity between paralogous mouse and human homeobox genes

**DOI:** 10.1186/s13227-018-0091-4

**Published:** 2018-01-27

**Authors:** Amy H. Royall, Ignacio Maeso, Thomas L. Dunwell, Peter W. H. Holland

**Affiliations:** 1 Department of Zoology, University of Oxford, South Parks Road, Oxford, OX1 3PS UK; 2 Centro Andaluz de Biología del Desarrollo, Consejo Superior de Investigaciones Científicas/Universidad Pablo de Olavide, 41013 Seville, Spain

**Keywords:** Gene duplication, Gene loss, Homeodomain, PRD class, *ARGFX*, Transcription factor, Compensation, Blastocyst

## Abstract

**Background:**

ETCHbox genes are eutherian-specific homeobox genes expressed during preimplantation development at a time when the first cell lineage decisions are being made. The mouse has an unusual repertoire of ETCHbox genes with several gene families lost in evolution and the remaining two, *Crxos* and *Obox*, greatly divergent in sequence and number. Each has undergone duplication to give a double homeodomain *Crxos* locus and a large cluster of over 60 *Obox* loci. The gene content differences between species raise important questions about how evolution can tolerate loss of genes implicated in key developmental events.

**Results:**

We find that *Crxos* internal duplication occurred in the mouse lineage, while *Obox* duplication was stepwise, generating subgroups with distinct sequence and expression. Ectopic expression of three *Obox* genes and a *Crxos* transcript in primary mouse embryonic cells followed by transcriptome sequencing allowed investigation into their functional roles. We find distinct transcriptomic influences for different *Obox* subgroups and *Crxos*, including modulation of genes related to zygotic genome activation and preparation for blastocyst formation. Comparison with similar experiments performed using human homeobox genes reveals striking overlap between genes downstream of mouse *Crxos* and genes downstream of human *ARGFX*.

**Conclusions:**

Mouse *Crxos* and human *ARGFX* homeobox genes are paralogous rather than orthologous, yet they have evolved to regulate a common set of genes. This suggests there was compensation of function alongside gene loss through co-option of a different locus. Functional compensation by non-orthologous genes with dissimilar sequences is unusual but may indicate underlying distributed robustness. Compensation may be driven by the strong evolutionary pressure for successful early embryo development.

**Electronic supplementary material:**

The online version of this article (10.1186/s13227-018-0091-4) contains supplementary material, which is available to authorized users.

## Background

Members of the homeobox superclass are widespread across eukaryotes, and their encoded proteins function mainly as transcription factors [1]. The proteins contain a DNA-binding domain known as the homeodomain which is highly variable at the primary sequence level. In animals, homeobox genes can be assigned to eleven classes, containing ~ 100 gene families in humans [1]. The largest homeobox gene class in animals is the ANTP class, with the PRD class being second largest. Some homeobox genes are highly conserved throughout animals; others, such as the mammalian X-linked *Rhox* cluster, have been evolving rapidly through duplication and sequence divergence [2]. Some homeobox gene families have been lost from certain evolutionary lineages secondarily [3, 4].

Ten years ago, several PRD class homeobox genes with highly divergent sequences were identified in the human genome (*TPRX1*, *TPRX2*, *DPRX*, *LEUTX* and *ARGFX*); it is now known that these are specific to eutherian mammals and arose through duplication of an Otx family gene *Crx* [4–7]. Each of these genes has expression limited to the germ line and early embryos in humans, and collectively they have been named Eutherian Totipotent Cell Homeobox (ETCHbox) genes [5, 7, 8]. Ectopic expression of several human ETCHbox genes revealed roles in transcriptional regulation of genes that have a peak of expression in the eight-cell embryo and morula stage, around the time of embryo compaction and following embryonic genome activation in humans [7, 9].

**Fig. 1:**
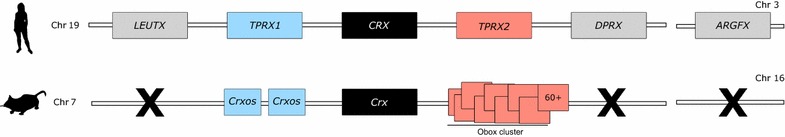
ETCHbox genes in human and mouse. The ETCHbox group of homeobox genes has six members: *Leutx*, *Tprx1*, *Tprx2*, *Dprx*, *Argfx* and *Pargfx*. In human, *Pargfx* has been lost in evolution. In mouse, only *Tprx1* and *Tprx2* orthologues remain and are referred to as *Crxos* and *Obox*, respectively; each has undergone sequence divergence and gene duplication

Until recently, these genes were thought to be lost from the mouse genome due to the absence of genes with highly similar homeobox sequences to human ETCHbox genes. The genomic regions syntenic to *TPRX1* and *TPRX2*, flanking the *Crx* gene on mouse chromosome 7, contain homeobox genes, but these are so distinct in sequence from the human genes that it was hypothesised they had arisen through independent tandem gene duplications in mouse ancestry [4, 10]. Each of these mouse genes, *Crx*-opposite strand (*Crxos*) [11] and oocyte-specific homeobox (*Obox*), have also undergone additional tandem duplication events not evident at the human locus. However, with increased sampling of rodents, the long phylogenetic branch lengths leading to *Crxos* and *Obox* could be broken and the mouse genes were shown to be highly divergent orthologues of *TPRX1* and *TPRX2*, respectively [7]. The mouse genome has no orthologous homeobox genes in loci syntenic to any other ETCHbox gene (Fig. 1; [7]). This unique genome organisation is quite different from human and most other mammals and raises important questions about the function of these genes in the mouse. For example, does extensive sequence divergence of the mouse genes imply they have acquired distinct functions from their human orthologues? What is the functional consequence of secondary duplications experienced by these genes in mice? With loss of *Dprx*, *Leutx*, *Pargfx* and *Argfx* in mice, have any compensatory mechanisms evolved such that *Crxos* or *Obox* genes take over the function of the lost genes?

Previous studies of mouse *Crxos* revealed that the locus gives rise to three distinct transcripts: a long transcript encompassing the two duplicate genes (and two homeoboxes) and one transcript from each duplicate. The complete *Crxos* locus has six exons with homeobox sequences spanning exons 2–3, and 5–6. The long transcript consists of exons 1, 2, 3 (partially), 5 and 6, with the shorter transcripts containing either exons 1, 2 and 3, or exons 4, 5 and 6 [12, 13]. Experiments involving ectopic expression in embryonic stem cells followed by quantification of candidate downstream targets have suggested that *Crxos* is involved in cell pluripotency with each shorter variant inhibiting differentiation markers [13–15]. Furthermore, an RNAi screen has identified *Crxos* as important for correct formation of the inner cell mass in the blastocyst and for hatching and outgrowth [16]. Another study has suggested that the long isoform is involved in specifying the primitive endoderm lineage [17].

The *Obox* genes were initially described as transcripts in unfertilised mouse eggs [18], and expression has now been reported throughout early murine development [19, 20]. Secondary duplication of *Obox* genes generated multiple loci. In a recent genome assembly (GRCm38/mm10), an array of five *Obox* loci is annotated, *Obox1*, *Obox2*, *Obox3*, *Obox5* and *Obox6*, with an additional locus *Obox7* given ‘provisional’ status. Previously, *Obox4* was annotated, but this is now listed as a ‘partial’ and unplaced annotation. Cheng et al. [19] showed that *Obox1* and *Obox2* have high sequence similarity and suggested they have the same expression pattern in the preimplantation embryo; they also found that *Obox1/2*, *Obox3* and *Obox5* are most highly expressed in the one-cell stage embryo with expression decreasing until no expression is detected in the morula. The *Obox6* locus, in contrast, has elevated expression between the two-cell and morula stages. When *Obox6* was knocked out in mice by homologous recombination, offspring exhibited normal development and were fertile [19]. More recent genome exploration has revealed that the *Obox* duplications were far more extensive than previously recognised and have generated over 60 distinct loci [7]. Not all loci have complete homeobox sequences, but it is clear that the diversity of *Obox* genes had been vastly underestimated. This raises new questions regarding the function of the *Obox* cluster in the light of these extensive duplications.

In this study, we use ectopic expression and transcriptomic analysis to investigate the function of mouse ETCHbox genes (*Crxos* and *Obox*). Our goals were to investigate whether these divergent genes have similar or distinct developmental functions to their human homologues, and whether *Crxos* or *Obox* genes have taken over roles associated with ETCHbox genes secondarily lost in mouse evolution. We find that ectopic expression of *Crxos* or *Obox* genes in cultured mouse embryonic fibroblasts induces large transcriptomic changes which can be related to notable events in the preimplantation embryo. We also argue that *Crxos* functions have evolved to compensate partially for loss of the *Argfx* homeobox gene.

## Results

### Sequence and expression diversity within expanded mouse ETCHbox clusters

The orthologues of both *TPRX1* (*Crxos*) and *TPRX2* (*Obox*) genes are duplicated in mouse. We previously identified 67 *Obox* loci (including pseudogenes) clustered on chromosome 7 in the region syntenic to the *TPRX2* locus in humans [7]. To investigate the pathway of duplication and search for groupings of highly similar loci, we conducted phylogenetic analyses using *Obox* nucleotide sequences (Additional files 1A and 2). One locus previously identified was found to not contain a homeodomain or high sequence similarity to annotated *Obox* genes. This sequence was therefore disregarded resulting in a total of 66 loci. Phylogenetic analysis revealed three main categories, named here OboxA, OboxB and OboxD (Additional file 1A) containing 13, 26 and 26 loci, respectively, arranged in an interspersed manner (Additional file 1B). These include putatively functional loci and pseudogenes. Since there are many more loci than previously named, we propose a new Obox nomenclature system based on DNA sequence (see Additional file 1C). We identify one additional locus that does not fit within these main groups, *Oboxc*.

**Fig. 2:**
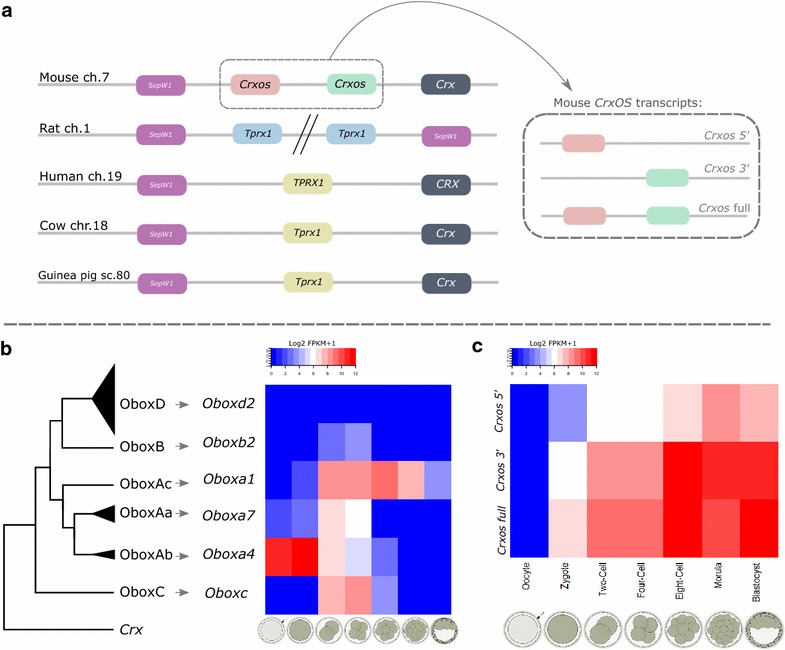
ETCHbox organisation and expression in preimplantation development: **a** The *Crxos* locus has undergone a mouse-specific duplication and generates three transcripts: two with a single homeobox and a composite double-homeobox transcript. *Crxos* is the *Tprx1* orthologue although originally named as a distinct gene. **b** Analysis of protein and nucleotide sequences of 66 *Obox* genes in mouse identified four groupings; sequences and expression profiles enable OboxA to be split into three subgroups. **c** The three *Crxos* transcripts have the same preimplantation temporal expression pattern, with the 3′ single-homeobox transcript generally having higher RNA levels

Of these 66 *Obox* loci, we suggest that 28 have potential to be translated into protein sequences with a full homeodomain (Additional file 2). These deduced protein sequences were then used in a second phylogenetic analysis which indicated three subgroups within the OboxA group (Table 1; Fig. 2b; Additional file 1D). Analysis of RNA sequencing data from preimplantation mouse embryos revealed that temporal expression is similar within *Obox* groups or subgroups, but can be subtly different between them (Fig. 2b). Expression of the vast majority loci within the OboxD group was not detected in the early embryo, with the exception of *Oboxd10* which shows low-level expression (FPKM 4.09) at the two-cell stage (Table 1). Overall, we identified four expression profiles: oocyte to two cell (OboxAb), two cell to four cell (OboxAa, OboxB), two cell to eight cell (OboxC) and two cell to blastocyst (OboxAc) (Fig. 2b). The observed expression patterns for *Oboxa1*, *Oboxa2*, *Oboxa3*, *Oboxa4* and *Oboxa6* are consistent with previous studies [18–20]. Based on sequence and expression similarity, we hypothesise there is likely to be functional redundancy within, but not usually between, *Obox* groups and subgroups.

**Table 1:** Groupings of mouse *Obox* genes with corresponding expression patterns

Human orthologue	Group	Subgroup	Members with ORF and HD	Members expressed in embryo	Example	Expression
*TPRX2*	OboxA	OboxAa	5	5	*Oboxa7*	Two cell to four cell
		OboxAb	4	4	*Oboxa4*	Oocyte to two cell
		OboxAc	1	1	*Oboxa1*	Two cell to morula
	OboxB	OboxB	1	1	*Oboxb2*	Two cell to four cell
	OboxC	OboxC	1	1	*Oboxc*	Two cell to eight cell
	OboxD	OboxD	16	1*	*Oboxd10*	Two cell
*TPRX1*	Crxos	NA	3	3	*Crxos*	Two cell to blastocyst

* Exact number uncertain because of 100% sequence similarity

The murine gene *Crxos*, orthologous to human *TPRX1*, has duplicated in tandem with the locus generating three distinct transcripts (Fig. 2a). To date the duplication, we used a conserved domain search to examine the genomic region between *Crx* and *Sepw1* loci (the location of *TPRX1*/*Crxos*) for homeobox sequences. In cow (*Bos taurus*), guinea pig (*Cavia porcellus*), naked mole rat (*Heterocephalus glaber*) and brown rat (*Rattus norvegicus*), only one homeobox was found (Fig. 2a, Additional file 1E), although *R. norvegicus* has an apparently independent duplication of the region containing extra copies of both *Crxos* and *Sepw1*. In contrast, using TBLASTN in the Algerian mouse (*Mus spretus*, Jackson laboratory strain #001146*)*, we found two homeobox sequences and regions homologous to all *M. musculus* exons (Additional file 1E and F). These data reveal that the internal duplication to generate a double-homeobox *Crxos* gene occurred in the ancestry of the *Mus* genus after the split of the mouse and rat lineages.

Analysis of RNA sequencing data from mouse preimplantation embryos revealed that the 3′ single-homeobox *Crxos* transcript has higher expression than the 5′ single-homeobox transcript, although the temporal profile is the same (Fig. 2c). The 3′ *Crxos* transcript has also been previously reported to encode sequences essential for nuclear localisation of the protein [21].

### Ectopic expression of *Crxos* and *Obox* genes induces transcriptomic changes

As homeobox genes encode transcription factors, we asked whether ectopic expression of mouse ETCHbox genes could induce transcriptional changes and how such changes are related to possible in vivo roles. Due to the complex genomic organisation, we used the above evolutionary analyses to inform the choice of genes. We selected three *Obox* genes with different expression patterns and from different subgroups to test the hypothesis that genes from different subgroups have distinct activities. We also selected the 3′ *Crxos* single homeodomain transcript as the most highly expressed splice variant of *Crxos;* this will now be referred to as ‘*Crxos’* throughout.

We expressed ectopically *Oboxa1* (subgroup OboxAc, *Obox6* in earlier nomenclature), *Oboxa4* (subgroup OboxAb, formerly *Obox1*), *Oboxa7* (subgroup OboxAa) or *Crxos* under a constitutive promoter in primary mouse embryonic fibroblasts and assayed transcriptome-wide effects through RNA sequencing. In each case, we determined catalogues of genes that were up- or down-regulated 48 h after ectopic expression (Additional file 3). These catalogues could include direct and indirect targets. To identify embryonic processes potentially influenced by *Crxos*, *Oboxa1*, *Oboxa4*, and *Oboxa7,* we examined whether each catalogue of up- or down-regulated genes had overlap with defined ‘temporal expression profiles’ (these are sets of genes grouped on the basis of expression pattern in mouse preimplantation development; shown in Additional files 1G and 4). We also used gene ontology (GO terms) to provide further functional information on groups of genes. The numbers of genes affected significantly by each treatment, and the overlaps between experiments are shown in Additional file 1H.

### *Crxos* up- and down-regulated profiles

**Fig. 3:**
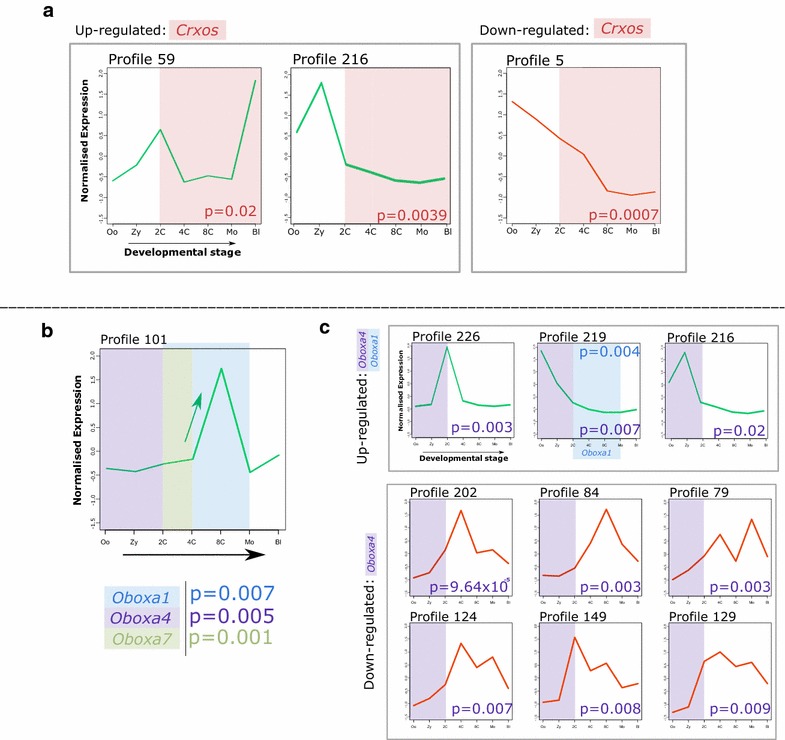
Enriched profiles following *Crxos* or *Obox* ectopic expression. Following over-expression of *Crxos*, *Oboxa1*, *Oboxa4 or Oboxa7* in cultured mouse embryonic cells, we identified genome-wide transcriptomic changes. Each set of up- or down-regulated genes was compared to sets of genes assigned to distinct temporal expression profiles to test for enrichment (Fisher’s exact test, corrected *p* values shown). **a** Genes up-regulated following *Crxos* ectopic expression are enriched for two profiles, 59 and 216, although we suggest profile 216 is an off-target effect. Genes down-regulated are enriched for profile 5. Pink shading indicates time of *Crxos* expression. **b** Genes in profile 101 are up-regulated by *Oboxa1*, *Oboxa4* and *Oboxa7*. Blue, purple or green shading indicates time of expression for *Oboxa1*, *Oboxa4* or *Oboxa7*, respectively. **c** Genes up-regulated by *Oboxa4* ectopic expression are enriched for two profiles not affected by other *Obox* genes (226, 216) and one profile also affected by *Oboxa1* (219). Genes down-regulated by *Oboxa4* ectopic expression are enriched for six profiles not affected by other *Obox* genes (202, 84, 79, 124, 149, 129). Green curves relate to up-regulated genes; red curves relate to down-regulated genes. Oo = oocyte, Zy = zygote, 2C = two-cell embryo, 4C = four-cell embryo, 8C = eight-cell embryo, Mo = morula, Bl = blastocyst

The catalogue of genes down-regulated following *Crxos* ectopic expression is enriched in genes from a single temporal expression profile, number 5 (Fisher’s test *p* = 0.0007; Fig. 3a, Additional file 5). Profile 5 contains genes with high levels of mRNA in the oocyte, which decrease as development proceeds (Fig. 3a). Genes up-regulated by *Crxos* ectopic expression are enriched for genes in profile 59 (Fisher’s test *p* = 0.02, Additional file 5) composed of genes with a sharp peak of expression in the two-cell embryo, then lower expression from four cell to morula, before a second, larger expression peak in blastocyst. There is also enrichment for profile 216, but this profile is not consistent with normal *Crxos* expression so may be off-target. Given that expression of *Crxos* begins in the two-cell embryo and extends to the blastocyst, we suggest that the second, larger peak of expression in profile 59 includes in vivo downstream targets of *Crxos* (Fig. 3a). Together, these results suggest that when *Crxos* transcription is initiated in the two-cell stage, it serves a role in preparing the embryo for blastocyst formation.

### *Obox* up- and down-regulated profiles

The catalogue of genes up-regulated by ectopic expression of *Oboxa1*, *Oboxa4* or *Oboxa7* showed extensive overlap. Each of these *Obox* genes elicited higher expression of genes found within temporal expression profile 101 (*Oboxa1 p* = 0.007, *Oboxa4 p* = 0.005, *Oboxa7 p* = 0.001; Fig. 3b, Additional file 5). In mouse development, genes in profile 101 are not expressed during the earliest stages of preimplantation development but show a sharp pulse of expression at the eight-cell stage. These genes are likely to have roles before the earliest cell fate decisions. The finding that three *Obox* genes, but not *Crxos*, elicit increased expression of similar sets of genes may be a consequence of the three closely related Obox proteins activating target genes through recognition of the same enhancer motifs [20]. Thus, after ectopic expression, an *Obox* gene may up-regulate targets usually regulated by a different *Obox* gene. Considering the expression profiles of *Oboxa1*, *Oboxa4* and *Oboxa7*, we suggest that genes within profile 101 are possible in vivo downstream targets of *Oboxa1* (OboxAc subclass) or *Oboxa7* (OboxAa subclass).

**Fig. 4:**
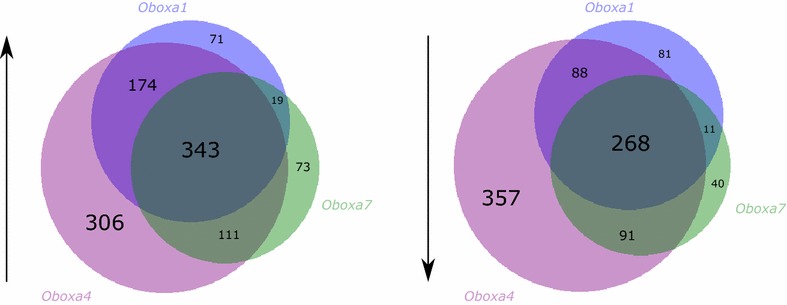
Overlap between genes downstream of *Obox* genes. Comparison of genes up- or down-regulated following ectopic expression of *Obox* genes; ectopic expression of the maternally expressed *Oboxa4* gene affects expression of additional downstream genes not affected by *Oboxa1* or *Oboxa7* expression

Considered across all temporal profiles, the three *Obox* genes examined up-regulate a common set of 343 genes and down-regulate a common set of 268 genes (Fig. 4). GO analysis reveals extracellular matrix (*p* = 4.9 × 10^−33^) and focal adhesion (*p* = 7.2 × 10^−8^) as enriched functions among the commonly up-regulated genes, and DNA replication (*p* = 1.7 × 10^−5^) enriched among the down-regulated genes (Additional file 1I).

The comparison of all downstream genes also reveals that ectopic expression of *Oboxa4* has the most distinct effects of the three *Obox* genes tested, affecting more than 600 genes not significantly affected by the other *Obox* genes (Fig. 4). The distinctiveness of *Oboxa4* is also evident at the level of temporal profiles. In addition to the shared profile 101, genes up-regulated following ectopic expression of *Oboxa4* were enriched for three further temporal profiles: 216, 219 and 226 (Fig. 3C, Additional file 5). The first two of these comprise genes with in vivo expression peaks in the zygote and two-cell embryo. Since *Oboxa4* is predominantly expressed as a maternal transcript, these profiles are consistent with being downstream in vivo direct or indirect targets (Fig. 3c). Genes down-regulated following *Oboxa4* ectopic expression are significantly enriched for six expression profiles, each of which has an mRNA peak after zygotic genome activation (ZGA; [22]): profiles 202, 84, 79, 124, 149, 129; collective Fisher’s test *p* = 2.7 × 10^−13^ (Fig. 3b, Additional file 5). These profiles, therefore, are consistent with being negatively regulated downstream in vivo targets, with maternal *Oboxa4* suppressing expression until after ZGA. A gene ontology (GO) analysis of the regulated genes in these profiles shows enrichment for ribosome biogenesis functions (*p* < 0.001). Profile 219 is also enriched in the genes up-regulated downstream of *Oboxa1*, although considering the temporal expression of *Oboxa1* in the embryo this is more likely to be an in vivo target of *Oboxa4* (Fig. 3c).

### Comparison between human and mouse ETCHbox gene functions

**Fig. 5:**
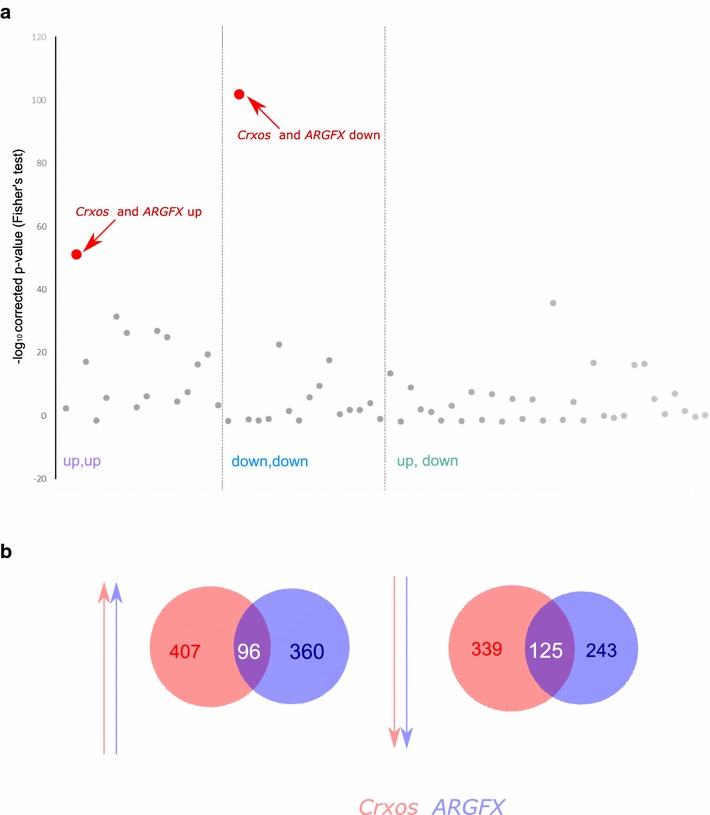
Similarity between genes downstream of human *ARGFX* and mouse *Crxos*. **a** The sets of genes up- or down-regulated following ectopic expression are compared between each mouse and human ETCHbox gene after filtering for one-to-one orthologues (human data from Ref. [7]); *y*-axis shows -log(*p* values) derived from pairwise Fisher’s exact test. Many comparisons are significant; the most striking similarities are between downstream targets of mouse *Crxos* and human *ARGFX* (up-regulated genes *p* = 3 × 10^−52^; down-regulated genes *p* = 4.2 × 10^−102^). Order of comparisons, from left to right, given in Additional file 6. **b** Proportional Venn diagrams showing extent of overlap between human and mouse one-to-one orthologues affected by *Crxos* ectopic expression in primary mouse embryonic fibroblasts and *ARGFX* in primary adult human fibroblasts

The evolutionary loss of *Argfx*, *Leutx* and *Dprx* ETCHbox genes in mouse, and duplication of the remaining *Tprx1* and *Tprx2* genes, raises questions about functional similarities and differences between mouse and human. We compared the sets of genes significantly up- and down-regulated after ectopic expression of human *TPRX1*, *ARGFX*, *DPRX* and *LEUTX* [7] with each of the gene sets up- or down-regulated by mouse *Crxos*, *Oboxa1*, *Oboxa4* and *Oboxa7*. Two of these comparisons revealed striking overlap (Fig. 5a; Additional files 6 and 7). We found that for 96 of the genes up-regulated by *ARGFX* in human cells, their mouse orthologues were also up-regulated by *Crxos* in mouse cells (Fig. 5b; Fisher’s test *p* = 3 × 10^−52^; Additional file 7). Similarly, the set of genes down-regulated by *ARGFX* in human cells showed significant similarity to the set of genes down-regulated by *Crxos* in mouse cells (Fig. 5b; 125 one-to-one orthologues, *p* = 2.4 × 10^−102^; Additional file 7). Of these, 98% of the jointly down-regulated orthologues are expressed in the mouse preimplantation embryo, as are 50% of the jointly up-regulated genes. These data suggest close similarity of function between human *ARGFX* and mouse *Crxos*, despite these being non-orthologous proteins. The direct orthologue of mouse *Crxos* is human *TPRX1*, not *ARGFX*; these orthologous genes seem to have contrasting functions following ectopic expression since many genes down-regulated by *Crxos* over-expression are up-regulated by *TPRX1* (*p* = 1.58^−36^; Additional file 6). There are also additional significant overlaps observed between genes (Fig. 5a), suggesting further overlapping functions of human and mouse ETCHbox genes.

### Targets with strong response are associated with embryonic milestones

The analysis of function using temporal gene profiles pays equal attention to genes with low and high expression and does not distinguish between mildly or strongly up- and down-regulated targets. We therefore asked which target genes were most strongly affected by ectopic expression of mouse *Crxos* and *Obox* genes (highest fold change up-regulated in Table 2; highest fold change down-regulated Additional file 1J). Three of the 10 most strongly up-regulated genes following *Crxos* ectopic expression have functions related to the extracellular matrix (ECM). Similarly, when ordered by fold change, three of the genes up-regulated most strongly following *Oboxa1* and *Oboxa4* ectopic expression are ECM components or function as mediators of ECM communication: *Col8a2 (Oboxa1* and *Oboxa4)*, *Eln (Oboxa1* and *Oboxa4)*, *Egfl6 (Oboxa1)* and *Aoc3 (Oboxa4).*

**Table 2:** Genes with highest up-regulated expression fold change following ectopic expression of mouse ETCHbox genes

Rank	*Oboxa1*		*Oboxa4*		*Oboxa7*		*Crxos*	
	Gene	Log_2_ fold change	Gene	Log_2_ fold change	Gene	Log_2_ fold change	Gene	Log_2_ fold change
1	*Lgr5*	2.02	*Megf6*	2.24	*Fmo1*	2.45	*Apod*	1.85
2	*Megf6*	1.82	*Lgr5*	1.91	*Megf6*	1.65	*Serpina3* *g*	1.64
3	*Myh1*	1.66	*Myh1*	1.87	*Myh11*	1.58	*Tnn*	1.56
4	*Myh11*	1.54	*Eln*	1.78	*Egfl6*	1.57	*Megf6*	1.53
5	*Col8a2*	1.48	*Gja5*	1.78	*Cdo1*	1.55	*Cxcl14*	1.53
6	*Eln*	1.47	*Gdpd2*	1.71	*Rbp3*	1.44	*Mfap4*	1.53
7	*Gja5*	1.38	*Col8a2*	1.71	*Apoe*	1.39	*Dpt*	1.52
8	*Egfl6*	1.38	*Gucy1a3*	1.69	*Chit1*	1.35	*Egfl6*	1.49
9	*Itga11*	1.36	*Aoc3*	1.68	*Gja5*	1.32	*Ch25* *h*	1.48
10	*Epha3*	1.36	*Hmcn1*	1.64	*Slpi*	1.31	*Serping1*	1.43

The most strongly down-regulated genes have functions in processes distinct from the up-regulated genes (Additional file 1J). Among the top 10 down-regulated genes following *Oboxa1* ectopic expression is *Sox2*, a transcription factor involved in specification of the inner cell mass of the blastocyst and deployed in iPS cell production as one of the Yamanaka factors [23]. Similarly, a gene strongly down-regulated after *Crxos* ectopic expression is implicated in trophectoderm specification and development, *Slco2a1* (log_2_ fold change of − 0.94) [24, 25]. The effect of ectopic ETCHbox expression on other genes with known roles in preimplantation development is given in Additional file 1K.

## Discussion

Mammalian preimplantation development encompasses several processes common to a wide range of species, such as generation of a hollow blastocyst and implantation into maternal endometrial tissue. Mice have long been used judiciously as models for human embryogenesis [26–29], with caution urged about extrapolation between species [30]. Furthermore, consistent with the hourglass model which describes propensity for evolutionary change early and late in development, transcriptomic analyses have highlighted variation in the earliest stages of development between different mammalian species [31, 32]. Similarly, several of the ETCHbox genes implicated in regulation of human preimplantation gene expression have been secondarily lost in mice [7]. These findings raise questions about how far pathways and regulatory networks discovered in mice can be applied to human preimplantation development and vice versa. We have studied the mouse ETCHbox genes which, like their human orthologues, are expressed specifically in the preimplantation embryo but which have very different numbers and genomic composition to humans. Extensive gene loss, sequence divergence and duplication of the remaining genes in mouse allowed us to investigate how differences at the genomic level relate to species-specific differences or similarities in preimplantation development.

In mouse, four of the six ancestral ETCHbox gene families have been lost leaving orthologues of just *Tprx1* and *Trpx2*. The mouse *Crxos* gene is the orthologue of human *TPRX1*. *Crxos* has duplicated and is processed to give three transcripts with common temporal expression profiles in the preimplantation mouse embryo. The 66 *Obox* loci are orthologous to human *TPRX2* and can be divided into four groups and six subgroups, with expression profiles mirroring molecular phylogenetic classification. The concordance between sequence and expression suggests there may be functional redundancy within *Obox* subgroups during preimplantation mouse development.

To investigate downstream activities and functional similarity to human genes, we ectopically expressed three *Obox* genes from different subgroups and the most highly expressed *Crxos* transcript in mouse embryonic fibroblasts. It could be argued that these cell types are very different from cells of the preimplantation embryo, but to conduct evolutionarily meaningful comparisons with data previously obtained using human fibroblasts [7] it is important to use comparable cell types. Additionally, ectopic expression of ETCHbox genes in primary cells is comparable to reprogramming experiments, which commonly use primary fibroblasts as the initial cell population.

We found that each of the genes elicited transcriptomic changes related to gene expression profiles of preimplantation stages, suggesting we have partially recapitulated the in vivo roles of mouse ETCHbox genes. For example, we find that over-expression of *Crxos* modifies the transcriptome of embryonic fibroblasts to partially mimic the transcriptome of the blastocyst, down-regulating transcripts that fall in abundance during cleavage stages and up-regulating genes with a blastocyst peak. These results are exciting since the blastocyst is composed of the descendent cell types from the first cell lineage decision: trophectoderm and pluripotent inner cell mass. It is also the time when the embryonic secretome communicates with maternal tissue before subsequent blastocyst invasion into the endoderm [27]. A role in formation of these cell types is emphasised by the finding that orthologues of the genes most strongly up-regulated by *Crxos* are implicated in implantation in human development: *Apod* and *Serpina3* (Table 2).

**Fig. 6:**
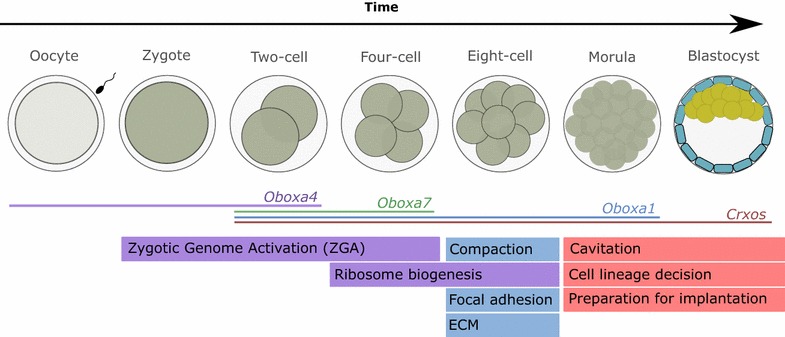
Cellular and embryonic processes potentially regulated by mouse ETCHbox genes. Global transcriptomic changes elicited by ectopic expression, and the embryonic expression profiles of ETCHbox genes themselves, highlight possible developmental milestones regulated by ETCHbox genes. We suggest that *Crxos* is involved in preparing the embryo for the first cell fate decision prior to the early blastocyst stage. *Obox* genes likely regulate a range of biological processes in vivo; we suggest *Oboxa4* is involved with early milestones including induction of zygotic gene expression, whereas *Oboxa1* and *Oboxa7* are involved in later events such as embryo compaction. Expression of the ETCHbox genes tested is represented by coloured lines (*Crxos* red, *Oboxa1* blue, *Oboxa4* purple, *Oboxa7* green)

Similarly, over-expression of *Oboxa4* caused transcriptomic changes mirroring those of the early embryo. *Oboxa4* is expressed maternally in the oocyte, and high levels of RNA are detected in the zygote. Ectopic expression of this gene caused down-regulation of genes that are not detectable as RNA in the earliest developmental stages, and up-regulation of those that are. In mouse ontogeny, zygotic genome activation (ZGA) is initiated earlier than in humans, with an early major wave of activation at the 2-cell stage and further waves of activation occurring until the morula stage [33]. The expression profiles of genes downstream of *Oboxa4* suggest that it may have a role in delaying or suppressing expression of embryonic genes, affecting timing of ZGA (Fig. 6).

For all *Obox* genes tested, we detect evidence of regulation of genes expressed in the eight-cell embryo and thus substantially earlier than the effect driven by *Crxos* ectopic expression. However, because this effect is seen for three different *Obox* genes, this may partially reflect cross-regulation between targets of closely related genes. On the basis of the normal expression profiles of *Obox* genes, we suggest the common effect is most likely to reflect in vivo function of *Oboxa1* or *Oboxa7* (Table 1). The eight-cell stage of mouse development, when many putative *Obox* target genes are expressed, is a particularly interesting developmental period. At this stage, the cells of the embryo increase their cell–cell contacts and elongate, and the embryo compacts in a critical morphological change which prepares the embryo for implantation (Fig. 6) [34–36]. Functions of genes affected include interactions with the ECM, adhesion to the external environment and DNA replication.

**Fig. 7:**
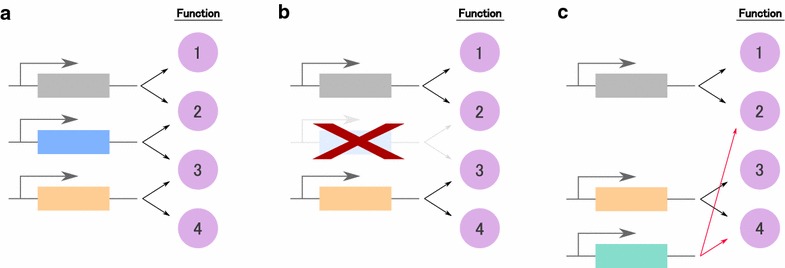
Simplified scenario for the evolution of functional redundancy. **a** A group of genes contribute to several biological processes with partial overlap of functions. **b** Partial functional overlap permits gene loss to persist as a temporary state. **c** Redundancy can be restored through gene duplication and divergence. The temporal sequence of (**b**) and (**c**) can be reversed. Partial redundancy affords buffering against somatic failure of a gene or process

One of the most striking findings of this study was the discovery of highly significant overlap between the set of genes up- and down-regulated by ectopic mouse ETCHbox genes with the set of genes affected by ectopic expression of human ETCHbox genes. We find many comparisons show significant overlaps (Fig. 5a), which may reflect widespread overlapping functions between the majority of ETCHbox genes. Most notably, many of the inferred downstream targets of *Crxos* in mouse are orthologous to the inferred downstream targets of *ARGFX* in human (96 orthologues commonly up-regulated, 125 commonly down-regulated). Much smaller overlap was detected for other pairs of mouse and human ETCHbox genes. There are differences between the biological activities of the two genes, however. For example, expressing *ARGFX* in human fibroblasts induced transcriptional changes that mirror a pulse of expression in the eight-cell human embryo [7]. In contrast, *Crxos*-induced transcriptional changes more closely parallel changes occurring at the blastocyst stage. Furthermore, *Crxos* has broader expression in mouse preimplantation development than does *ARGFX* in human. Together, these data suggest that before or after the loss of *ARGFX* in murid evolution, the *Crxos* gene took over roles originally undertaken by *ARGFX* and has also acquired additional targets and biological functions (Fig. 7). It is particularly intriguing that this functional compensation involved deployment of a paralogous rather than an orthologous homeobox gene. Indeed, *Crxos* seems to have contrasting transcriptomic effects to its direct orthologue *TPRX1*, with significant overlap between genes down-regulated by *Crxos* and those up-regulated by *TPRX1* (*p* = 1.58^−36^, Additional file 7).

The convergence of function between human and mouse ETCHbox genes provides an intriguing example of compensation and refinement of gene function alongside dynamic lineage-specific gene loss and expansions (Fig. 7). Such compensation would be favoured if the system displays ‘distributed robustness’ as defined by Wagner [37]. Under this model, individual genes may have overlapping rather than redundant functions, such that collectively the system has multiple routes to achieve the same endpoints. Extrapolating, we suggest that a variable set of ETCHbox genes regulates a suite of functions in eutherian preimplantation embryos that are shared between species, regardless of the particular repertoire of ETCHbox genes retained in a genome.

## Conclusions

The evolutionary history of ETCHbox genes, with extensive gene duplication, loss and sequence change during mammalian radiation, contrasts sharply to that of most other homeobox genes. We show that ectopic expression of these genes in mouse embryonic fibroblasts elicits transcriptomic changes that mirror transcriptomic changes in early development. For example, the maternally expressed *Oboxa4* gene can reduce expression of genes normally active after ZGA, other *Obox* genes elevate expression of genes that peak at the eight-cell stage, and *Crxos* expression causes transcriptomic changes mirroring pre-blastocyst development. Most strikingly, we find close similarity between the genes downstream of mouse *Crxos* and human *ARGFX*, despite these not being orthologous homeobox genes. These results point to an evolutionarily labile system where a set of regulatory genes can duplicate and be lost in evolution, but shift functionally to compensate for gene loss. Functional replacement by a paralogous gene might be favoured when evolution builds an inherently redundant system to ensure the robustness of critical developmental events.

## Methods

### Embryonic expression and phylogenetic analyses

To examine endogenous expression patterns, RNA sequencing files were acquired from the SRA database (SRA identifier SRP034543; Additional file 8) and aligned to the GRCm38/mm10 *M*. *musculus* genome using the STAR alignment tool [38]. The Cufflinks tool was used to extract FPKM expression values (Additional file 9). To investigate evolutionary relationships between genes, maximum likelihood phylogenies were generated from nucleotide or deduced amino acid sequences using RAxML with 500 bootstraps [39].

### Ectopic gene expression

Mouse embryonic fibroblasts (Sciencell #M7540-57) were cultured in Dulbecco’s modified Eagle medium (DMEM, ThermoFisher #41966-029) supplemented with 10% foetal calf serum (FCS) and 1% Pen–Strep in 5% CO_2_ at 37 °C on 0.001% poly-l-lysine-coated plasticware. Coding sequences of *Oboxa1*, *Oboxa4*, *Oboxa7* and *Crxos* were obtained from GenScript and cloned into a vector co-expressing daGFP; genes were C-terminally tagged with V5 and under control of a CMV promoter (Oxford Genetics #OG244). For ectopic expression, 10^6^ cells in 100 µl Opti-Mem (Gibco 11058-021) were electroporated (NEPA GENE; poring pulse: 175 volts, 5 ms, 4 pulses) with 10 µg of plasmid DNA and seeded in DMEM, 10% FCS, 1% Pen–Strep antibiotic (ThermoFisher #15140-122). Media was changed after 24 h and cells collected for FACS sorting after 48 h to enrich for transfected cells. RNA was extracted using the RNeasy microkit (Qiagen) and quality checked on an Experion electrophoresis station (Bio-Rad). RNA sequencing was performed on three replicates from each experimental condition and control (empty vector transfection); libraries were prepared using Illumina TruSeq, and paired-end RNA sequencing reads generated on the Hi-Seq 4000 platform (Oxford Genomics Centre), yielding 38.2–58.2 million reads per sample. Sequence reads were aligned to the GRCm38/mm10 *Mus musculus* genome using the STAR alignment tool, and gene expression levels for protein-coding genes assessed according to NCBI annotations (ftp://ftp.ncbi.nlm.nih.gov/genomes/Mus_musculus/ accessed 13 July 2016, FPKM read-outs in Additional file 9). The ectopically expressed genes were expressed at a level (assessed by FPKM) comparable to their embryonic expression levels (Additional file 1L).

### Differential gene expression analysis

DESeq2 was used to identify genes differentially expressed in response to homeobox gene transfection following FeatureCounts to retrieve raw read counts (Additional file 10) [40]. Experiments were matched by date to minimise the effects of day-to-day variation. The Cufflinks tool was used to estimate mean FPKM expression values (Additional file 11). Lists of up- and down-regulated genes for each experimental condition were produced using criteria of *p* < 0.05 (with Benjamini–Hochberg correction) and expression fold change greater than 1.25 or − 1.25 (Additional file 3). Transcripts with an FPKM > 2 were considered to be actively expressed.

To generate sets of genes with similar temporal expression profiles across preimplantation mouse development (oocyte, zygote, two cell, four cell, eight cell, morula and blastocyst), we took mouse RNAseq data processed as above, filtered to identify genes with a FPKM variance > 5 across preimplantation development, and clustered these into profiles using Mfuzz [41, 42]. This analysis generated 150 initial temporal profiles of gene expression. Sets of profiles with a correlation coefficient of over 0.95 were merged into composite clusters which are identified by IDs > 200 (Additional files 1G and 4). When high correlation coefficients were not all reciprocal within a set of profiles, an expression dendrogram was used to guide the process of merging.

### Profile enrichment

Pearson’s Chi-square test was used to test the null hypothesis that the number of differentially expressed genes is proportional across all temporal profiles. If the proportions are not equal between profiles (*p* < 0.05), Pearson’s statistic was used to identify the contribution of each profile to the overall difference. After removal of profiles inferred to contribute to the difference, Fisher’s exact test was used to verify that the differentially expressed genes were proportionally distributed between the remaining profiles and to find statistical difference between occupation of enriched and non-enriched profiles.

## Additional files

**Additional file 1.** (A) *Obox* phylogeny using nucleotide sequences. (B) Genomic organisation of *Mus musculus* ETCHbox genes. (C) Proposed new *Obox* nomenclature. The term ‘partial’ indicates an Obox sequence lacking a complete homeobox, and the term is not part of proposed nomenclature. (D) *Obox* phylogeny using protein sequences that contain a complete homeodomain. (E) Summary of number of homeoboxes in *Tprx1* syntenic region in different species. * In rat, the two homeobox sequences reflect an independent regional duplication. (F) Alignment of *M. musculus* and *M. spretus Tprx1*/*Crxos* homeodomains. (G) Normalised expression graphs of each temporal profile generated by Mfuzz. (H) Venn diagrams shown numbers of genes affected significantly by each treatment, and overlaps between experiments. (I) Enriched GO terms for genes affected by *Obox* ectopic expression. (J) Genes most strongly down-regulated following ectopic expression. (K) Effect of ectopic ETCHbox expression on selected genes with notable preimplantation or stem cell functions. (L) In vivo expression levels of ETCHbox genes at mouse preimplantation stages compared to ectopic expression levels of transfected genes.

**Additional file 2.** Nucleotide and protein sequences used for *Obox* phylogenetic analyses.

**Additional file 3.** Lists of genes significantly up- or down-regulated following ectopic expression.

**Additional file 4.** Mfuzz clusters and genes within each cluster.

**Additional file 5.** Observed and expected numbers of significantly regulated genes populating temporal expression profiles used for enrichment analysis.

**Additional file 6.** List of one-to-one orthologues similarly regulated following ectopic expression of human *ARGFX* and mouse *Crxos*.

**Additional file 7.** Data used for statistical tests of overlap between sets of one-to-one orthologues regulated by mouse and human ETCHbox genes.

**Additional file 8.** SRA data information used to generate embryonic gene expression data.

**Additional file 9.** Gene expression (FPKM) across mouse embryonic stages.

**Additional file 10.** Gene expression (raw reads) from triplicate RNA sequencing of mouse embryonic fibroblasts following ectopic expression of mouse ETCHbox genes.

**Additional file 11.** Average FPKM values for experimental datasets.

## Abbreviations

CMV: cytomegalovirus
DMEM: Dulbecco’s modified Eagle medium
ECM: extracellular matrix
FACS: fluorescence-activated cell sorting
FCS: foetal calf serum
FPKM: fragments per kilobase of transcript per million mapped reads
NCBI: National Center for Biotechnology Information
SRA: sequence read archive
ZGA: zygotic genome activation
