# Supplementary material for: Mouse Obox and Crxos modulate preimplantation transcriptional profiles revealing similarity between paralogous mouse and human homeobox genes
Source: EvoDevo. 2018 Jan 27;9:2. doi: 10.1186/s13227-018-0091-4 (PMC5787275; doi:10.1186/s13227-018-0091-4)
Supplement: Supplementary file 1 — Additional file 1. (A) Obox phylogeny using nucleotide sequences. (B) Genomic organisation of Mus musculus ETCHbox genes. (C) Proposed new Obox nomenclature. The term ‘partial’ indicates an Obox sequence lacking a complete homeobox, and the term is not part of proposed nomenclature. (D) Obox phylogeny using protein sequences that contain a complete homeodomain. (E) Summary of number of homeoboxes in Tprx1 syntenic region in different species. * In rat, the two homeobox sequences reflect an independent regional duplication. (F) Alignment of M. musculus and M. spretus Tprx1/Crxos homeodomains. (G) Normalised expression graphs of each temporal profile generated by Mfuzz. (H) Venn diagrams shown numbers of genes affected significantly by each treatment, and overlaps between experiments. (I) Enriched GO terms for genes affected by Obox ectopic expression. (J) Genes most strongly down-regulated following ectopic expression. (K) Effect of ectopic ETCHbox expression on selected genes with notable preimplantation or stem cell functions. (L) In vivo expression levels of ETCHbox genes at mouse preimplantation stages compared to ectopic expression levels of transfected genes. [file 13227_2018_91_MOESM1_ESM.pdf]

A

All *Obox* nucleotide sequences:

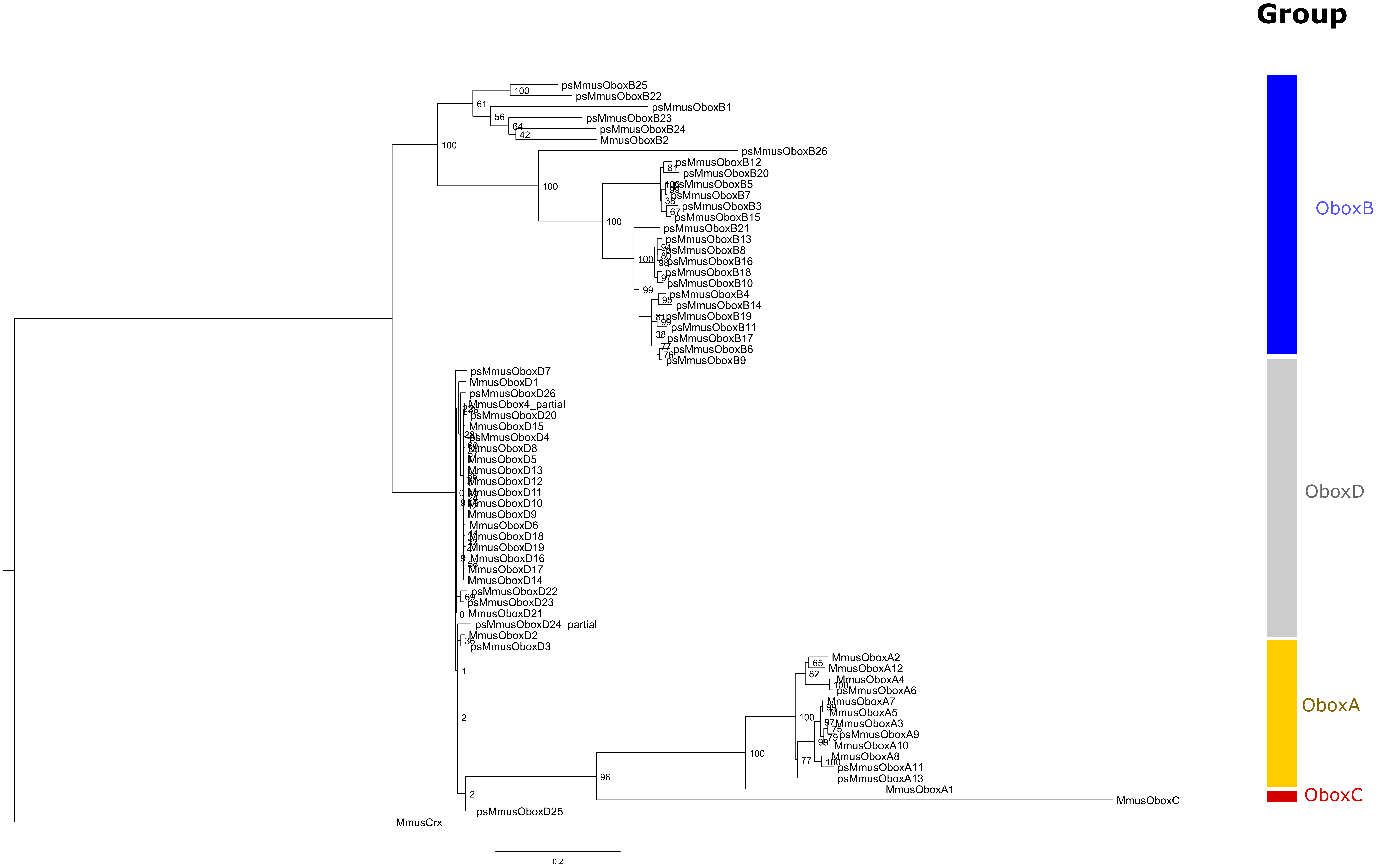

B

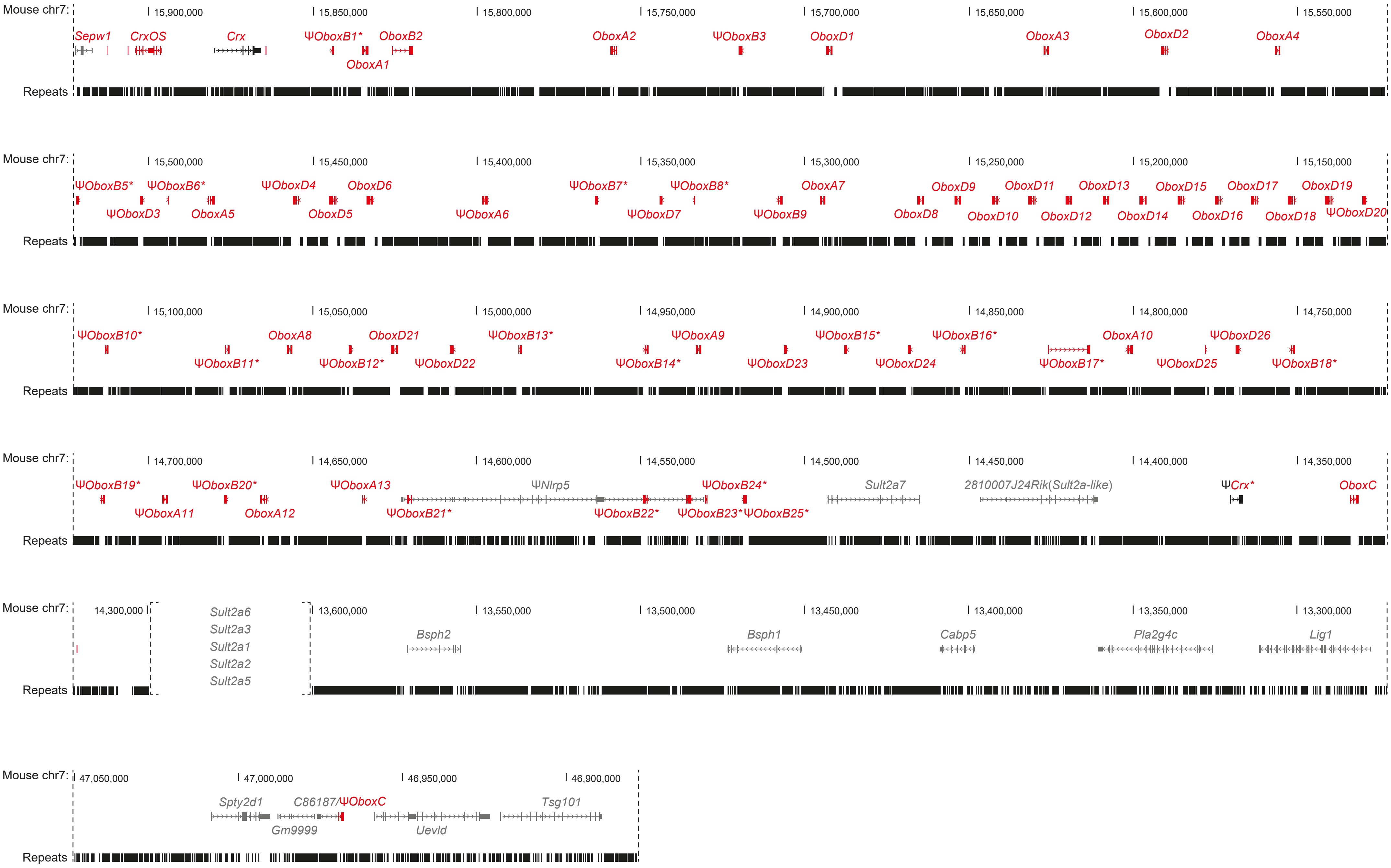
Mouse chr7:
15,900,000
15,850,000
15,800,000
15,750,000
15,700,000
15,650,000
15,600,000
15,550,000
Sepw1
CrxOS
Crx
ΨOboxB1*
OboxB2
OboxA1
OboxA2
ΨOboxB3
OboxD1
OboxA3
OboxD2
OboxA4
Repeats
Mouse chr7:
15,500,000
15,450,000
15,400,000
15,350,000
15,300,000
15,250,000
15,200,000
15,150,000
ΨOboxB5*
ΨOboxB6*
ΨOboxD4
OboxD6
ΨOboxB7*
ΨOboxB8*
OboxA7
OboxD9
OboxD11
OboxD13
OboxD15
OboxD17
OboxD19
ΨOboxD3
OboxA5
OboxD5
ΨOboxA6
ΨOboxD7
ΨOboxB9
OboxD8
OboxD10
OboxD12
OboxD14
OboxD16
OboxD18
ΨOboxD20
Repeats
Mouse chr7:
15,100,000
15,050,000
15,000,000
14,950,000
14,900,000
14,850,000
14,800,000
14,750,000
ΨOboxB10*
OboxA8
OboxD21
ΨOboxB13*
ΨOboxA9
ΨOboxB15*
ΨOboxB16*
OboxA10
ΨOboxD26
ΨOboxB11*
ΨOboxB12*
ΨOboxD22
ΨOboxB14*
ΨOboxD23
ΨOboxD24
ΨOboxB17*
ΨOboxD25
ΨOboxB18*
Repeats
Mouse chr7:
14,700,000
14,650,000
14,600,000
14,550,000
14,500,000
14,450,000
14,400,000
14,350,000
ΨOboxB19*
ΨOboxB20*
ΨOboxA13
ΨNlrp5
ΨOboxB24*
Sult2a7
2810007J24Rik(Sult2a-like)
ΨCrx*
OboxC
ΨOboxA11
OboxA12
ΨOboxB21*
ΨOboxB22*
ΨOboxB23*
ΨOboxB25*
Repeats
Mouse chr7:
14,300,000
Sult2a6
Sult2a3
Sult2a1
Sult2a2
Sult2a5
13,600,000
13,550,000
13,500,000
13,450,000
13,400,000
13,350,000
13,300,000
Bsph2
Bsph1
Cabp5
Pla2g4c
Lig1
Repeats
Mouse chr7:
47,050,000
47,000,000
46,950,000
46,900,000
Spty2d1
C86187/ΨOboxC
Tsg101
Gm9999
Uevld
Repeats

C

Alias	New nomenclature	Pseudogene?	Expressed in preimplantation Embryos?	Group
Gm5585/Obox8	*Oboxc*	N	Y	OboxC
Obox6	*Oboxa1*	N	Y	OboxA
Obox5	*Oboxa2*	N	N	OboxA
Obox3	*Oboxa3*	N	Y	OboxA
Obox1	*Oboxa4*	N	Y	OboxA
Gm5889/ obox3 ps6	*Oboxa5*	N	Y	OboxA
Obox2	*Oboxa6*	Y	N	OboxA
Gm8053/ obox3 ps5	*Oboxa7*	N	Y	OboxA
Gm8072/ obox3 ps4	*Oboxa8*	N	Y	OboxA
Gm8064/ obox3 ps3	*Oboxa9*	N	N	OboxA
Gm8040/obox3 ps2	*Oboxa10*	N	Y	OboxA
Gm8057/ obox3 ps1	*Oboxa11*	N	N	OboxA
Gm4745	*Oboxa12*	N	Y	OboxA
Gm18533 partial/obox3 ps7	*Oboxa13*	Y	N	OboxA
Gm4830/ obox3 ps8	--	--		Not analysed
--	*Oboxb1 partial*	Y	N	OboxB
Gm7235	*Oboxb2*	N	Y	OboxB
Gm20622/obox4 ps34	*Oboxb3*	Y	*N*	OboxB
Obox4 ps32	*Oboxb4*	Y	*N*	OboxB
--	*Oboxb5 partial*	Y	*N*	OboxB
--	*Oboxb6 partial*	Y	*N*	OboxB
--	*Oboxb7 partial*	Y	*N*	OboxB
Obox4 ps25	*Oboxb8 partial*	Y	*N*	OboxB
Gm20624 Obox4 ps24	*Oboxb9*	Y	*N*	OboxB
--	*Oboxb10*	Y	*N*	OboxB
Obox4 ps10	*Oboxb11*	Y	*N*	OboxB
--	*Oboxb12 partial*	Y	*N*	OboxB
--	*Oboxb13*	Y	*N*	OboxB
Obox4 ps7	*Oboxb14*	Y	*N*	OboxB
Gm27258/obox4 ps39	*Oboxb15 partial*	Y	*N*	OboxB
--	*Oboxb16*	Y	*N*	OboxB
Obox4 ps5	*Oboxb17*	Y	*N*	OboxB
--	*Oboxb18*	Y	*N*	OboxB
Obox4 ps4	*Oboxb19*	Y	*N*	OboxB
--	*Oboxb20 partial*	Y	*N*	OboxB
--	*Oboxb21*	Y	*N*	OboxB
Gm207180/Obox4 ps3	*Oboxb22*	Y	*N*	OboxB
--	*Oboxb23 partial*	Y	*N*	OboxB
Gm27172/Obox4 ps2	*Oboxb24 partial*	Y	*N*	OboxB
Gm27171/Obox4 ps1	*Oboxb25 partial*	Y	*N*	OboxB
--	*Oboxb26*	Y	*N*	OboxB
Obox4	*Obox4*	--	*N*	Not annotated
Gm4023/ obox4 ps33	*Oboxd1*	N	*N*	OboxD
Gm4018/obox4 ps31	*Oboxd2*	N	*N*	OboxD
Gm4016/obox4 ps30	*Oboxd3 partial*	Y	*N*	OboxD
Gm4014/ obox4 ps29	*Oboxd4*	Y	*N*	OboxD
Gm10678/obox4 ps28	*Oboxd5*	N	*N*	OboxD
Gm10679/obox4 ps27	*Oboxd6*	N	*N*	OboxD
Gm20623/ obox4 ps26	*Oboxd7 partial*	Y	*N*	OboxD
Gm4897/obox4 ps23	*Oboxd8*	N	*N*	OboxD
Gm4003/obox4 ps22	*Oboxd9*	N	*N*	OboxD
Gm4001/obox4 ps21	*Oboxd10*	N	*Y*	OboxD
Gm3998/obox4 ps20	*Oboxd11*	N	*N*	OboxD
Gm3996/obox4 ps19	*Oboxd12*	N	*N*	OboxD
Gm3994/obox4 ps18	*Oboxd13*	N	*N*	OboxD
Gm3989/obox4 ps17	*Oboxd14*	N	*N*	OboxD
Gm3987/obox4 ps16	*Oboxd15*	N	*N*	OboxD
Gm3684/obox4 ps15	*Oboxd16*	N	*N*	OboxD
Gm3981/obox4 ps14	*Oboxd17*	N	*N*	OboxD
Gm3980/obox4 ps13	*Oboxd18*	N	*N*	OboxD
Gm3977/obox4 ps12	*Oboxd19*	N	*N*	OboxD
Gm3968/obox4 ps11	*Oboxd20 partial*	N	*N*	OboxD
Gm3965/obox4 ps9	*Oboxd21*	N	*N*	OboxD
Gm3959/obox4 ps8	*Oboxd22 partial*	Y	*N*	OboxD
Gm20710/ obox4 ps6	*Oboxd23 partial*	N	*N*	OboxD
Gm20709/obox4 ps38	*Oboxd24 partial*	Y	*N*	OboxD
Gm20664/obox4 ps37	*Oboxd25 partial*	Y	*N*	OboxD
Gm20665/obox4 ps36	*Oboxd26 partial*	Y	*N*	OboxD
Gm7235/Obox4 ps35	--	--		Not annotated

D Protein-sequences with complete homeodomain:

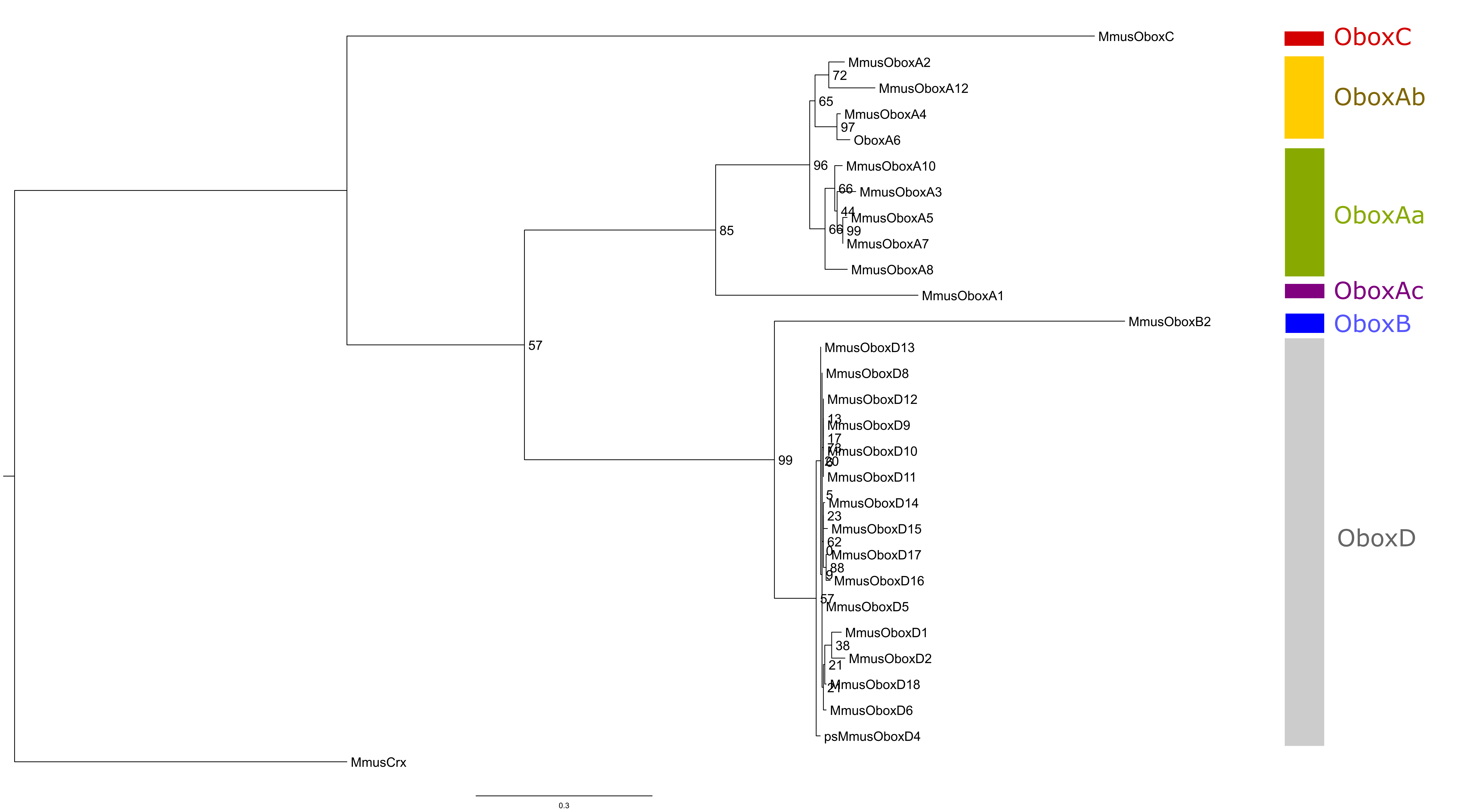

E

Species	Genome assembly	Number of homeodomains in *Tprx1* region
Algerian mouse	GCA_001624865.1	2
Naked mole rat	HetGla_female_1.0	1
Guinea pig	Broad/cavPor3	1
Cow	UMD 3.1.1	1
Rat	RGSC Rnor_6.0	2*

F

```
CLUSTAL O(1.2.4) multiple sequence alignment


CrxOS2spretus       MEEIDKMIIQMRLKDSKTVLISKTELTDLQFQKLRKHFETDRYPNEETLQAFAEELKLQK
CrxOS2musculus      MEEIDGMIVQMRLKDSKTVLISKTELTDEQFQKLRKHFETDRCPNEETLQAFAEELKLRK
CrxOS1spretus       MEAS------PRSLTSYTLGPLDQKFSWEQLWELEAYFKMEPYPDLQARKIMATRLKLKE
CrxOS1musculus      MEAS------PRSLTSCTLGPLDQKFSWEQLSELEAYFKVEPYPDLQDRKIMATRLKLKE
                    **         *   * *:   . :::  *: :*. :*: :  *: :  : :* .***::

CrxOS2spretus       AVIRPWFITQRHRMIGYRRLFMRYY-------------------RDWKTSREYSTTRRFD
CrxOS2musculus      DLIRSWFITQRHRMRGYRRLFMRYY-------------------RDWKTSREYSTTRSFD
CrxOS1spretus       EQVEAWFIQRSLEEEM-RPPLARLQQSARDDTPS--------------------------
CrxOS1musculus      EQVEAWFIQRSLEEEM-RPPLARLQQSALDGTSSPSHKALCCRPPSWKYRL-----IPIN
                      :. *** :  .    *  : *

CrxOS2spretus       RQKNSKECSQNDPGLPEALEALKRLKLSSGY-SRDGMSQDF
CrxOS2musculus      RQKNSKECSQNDPGLPEALEALKRLKLSSGYQSRDGMSQDF
CrxOS1spretus       -----------------------------------------
CrxOS1musculus      PPESSTSCKHSC-----------------------------
```

G

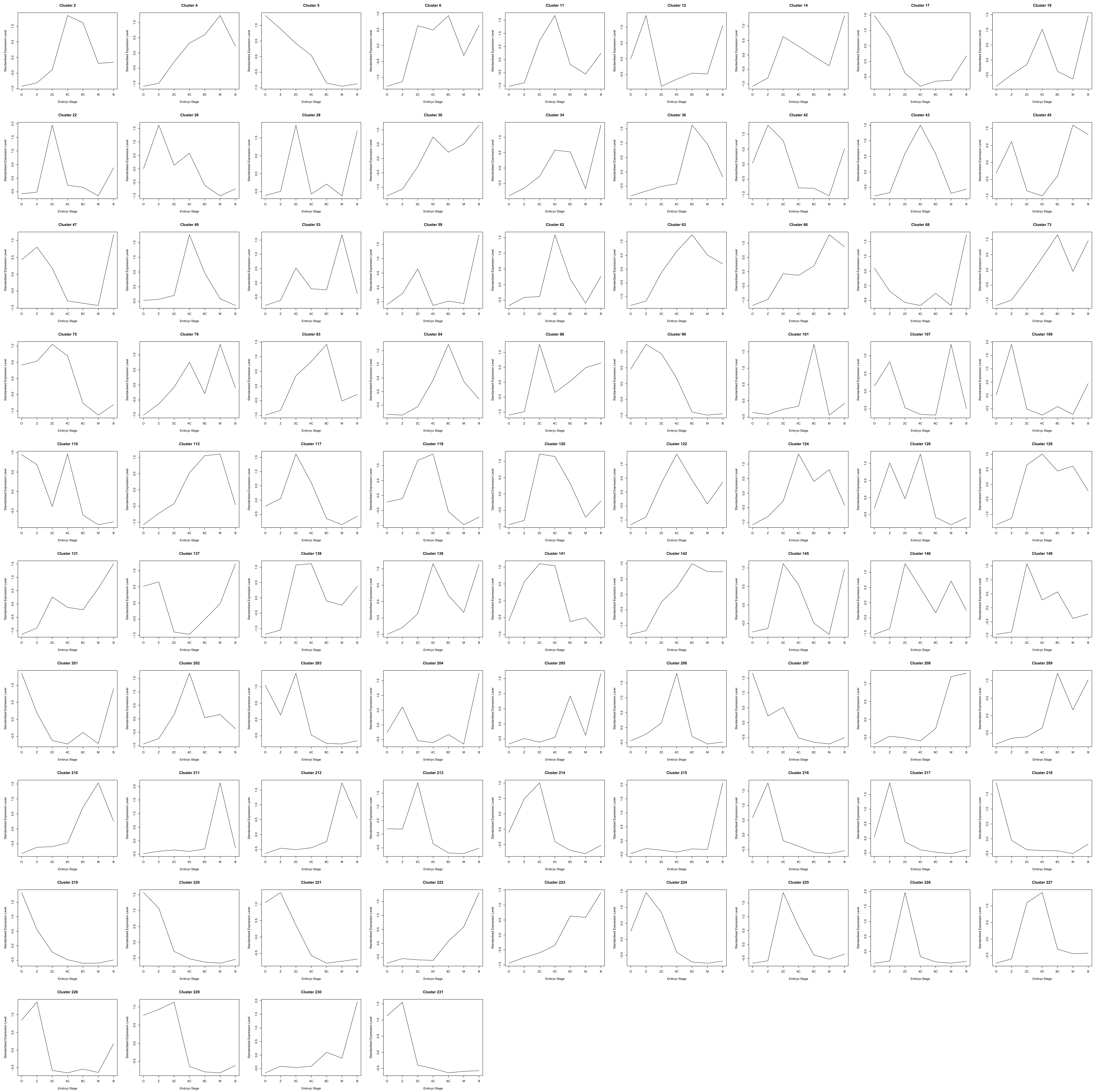
Cluster 2
Cluster 4
Cluster 5
Cluster 6
Cluster 11
Cluster 12
Cluster 14
Cluster 17
Cluster 19
Cluster 22
Cluster 26
Cluster 28
Cluster 30
Cluster 34
Cluster 36
Cluster 42
Cluster 43
Cluster 45
Cluster 47
Cluster 49
Cluster 53
Cluster 59
Cluster 62
Cluster 63
Cluster 66
Cluster 68
Cluster 73
Cluster 75
Cluster 79
Cluster 83
Cluster 84
Cluster 86
Cluster 99
Cluster 101
Cluster 107
Cluster 109
Cluster 110
Cluster 112
Cluster 117
Cluster 119
Cluster 120
Cluster 122
Cluster 124
Cluster 126
Cluster 129
Cluster 131
Cluster 137
Cluster 138
Cluster 139
Cluster 141
Cluster 142
Cluster 145
Cluster 146
Cluster 149
Cluster 201
Cluster 202
Cluster 203
Cluster 204
Cluster 205
Cluster 206
Cluster 207
Cluster 208
Cluster 209
Cluster 210
Cluster 211
Cluster 212
Cluster 213
Cluster 214
Cluster 215
Cluster 216
Cluster 217
Cluster 218
Cluster 219
Cluster 220
Cluster 221
Cluster 222
Cluster 223
Cluster 224
Cluster 225
Cluster 226
Cluster 227
Cluster 228
Cluster 229
Cluster 230
Cluster 231
Standardised Expression Level
Embryo Stage
O
Z
2C
4C
8C
M
B

H

Up-regulated following ectopic expression

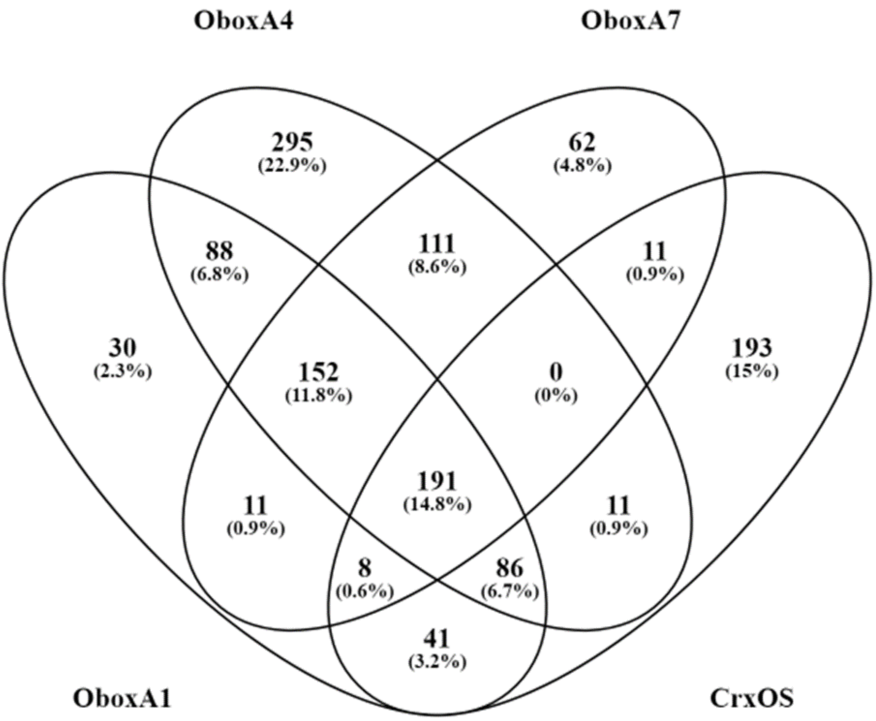

Down-regulated following ectopic expression

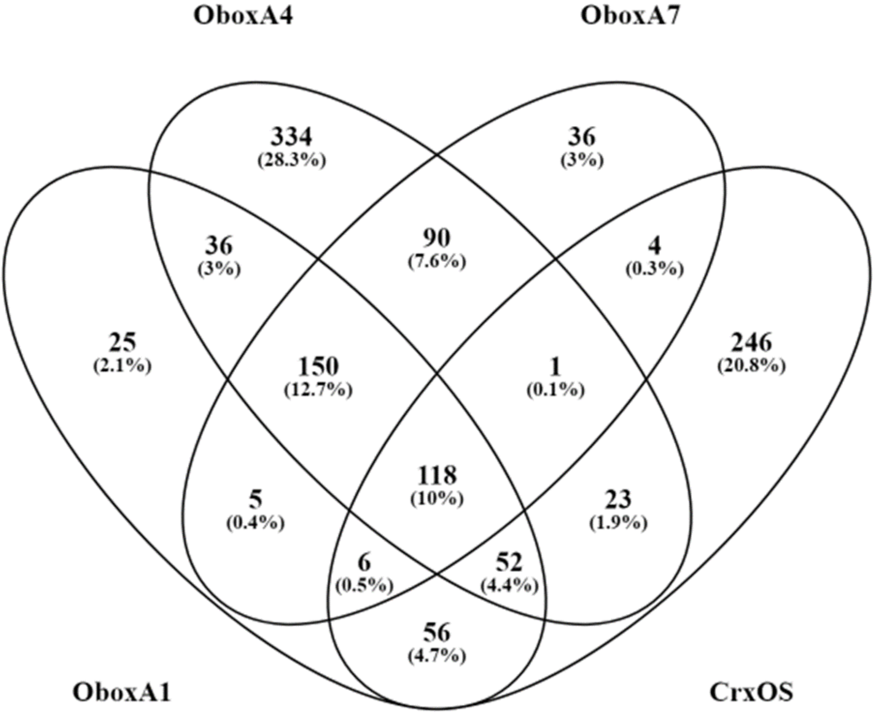

I

Obox jointly up-regulated following ectopic expression GO terms

Platform	Description	Count	P_Value	Benjamini
GOTERM_CC_DIRECT	**proteinaceous extracellular matrix**	52	2.00E-35	4.90E-33
UP_KEYWORDS	Glycoprotein	153	3.00E-34	7.30E-32
UP_KEYWORDS	Extracellular matrix	42	9.60E-32	1.20E-29
UP_KEYWORDS	Secreted	96	6.00E-31	5.00E-29
UP_KEYWORDS	Disulfide bond	131	3.80E-30	2.30E-28
GOTERM_CC_DIRECT	extracellular region	102	3.60E-30	4.40E-28
UP_SEQ_FEATURE	signal peptide	143	6.50E-31	8.00E-28
UP_KEYWORDS	Signal	157	7.60E-28	3.80E-26
UP_SEQ_FEATURE	glycosylation site:N-linked (GlcNAc...)	143	6.90E-25	4.20E-22
GOTERM_CC_DIRECT	extracellular matrix	40	5.90E-24	4.80E-22
UP_SEQ_FEATURE	disulfide bond	110	5.80E-21	2.30E-18
GOTERM_CC_DIRECT	extracellular space	64	6.90E-12	4.20E-10
GOTERM_CC_DIRECT	collagen trimer	15	8.80E-11	4.30E-09
INTERPRO	Thrombospondin, type 1 repeat	14	3.00E-11	9.60E-09
UP_KEYWORDS	Collagen	14	4.00E-10	1.10E-08
INTERPRO	Insulin-like growth factor binding protein, N-terminal	18	2.60E-11	1.70E-08
KEGG_PATHWAY	**Focal adhesion**	21	3.70E-10	7.20E-08
KEGG_PATHWAY	ECM-receptor interaction	14	3.10E-09	3.00E-07
INTERPRO	Collagen triple helix repeat	13	2.50E-09	5.40E-07
INTERPRO	EGF-like calcium-binding, conserved site	14	4.40E-09	7.00E-07
UP_KEYWORDS	EGF-like domain	18	4.90E-08	1.20E-06
UP_KEYWORDS	Hydroxylation	12	7.90E-08	1.80E-06
GOTERM_MF_DIRECT	extracellular matrix structural constituent	10	2.40E-08	5.30E-06
INTERPRO	EGF-type aspartate/asparagine hydroxylation site	13	4.90E-08	6.30E-06
GOTERM_BP_DIRECT	collagen fibril organization	10	1.60E-08	7.60E-06
INTERPRO	EGF-like, conserved site	17	1.10E-07	9.80E-06
SMART	TSP1	12	6.20E-08	1.00E-05
INTERPRO	EGF-like calcium-binding	14	1.10E-07	1.10E-05
INTERPRO	Epidermal growth factor-like domain	18	2.60E-07	2.10E-05
GOTERM_BP_DIRECT	anterior/posterior pattern specification	14	6.60E-08	2.50E-05
INTERPRO	Fibrillar collagen, C-terminal	6	4.10E-07	2.90E-05
SMART	COLFI	6	1.60E-06	8.60E-05
SMART	EGF_CA	14	2.20E-06	8.80E-05
UP_SEQ_FEATURE	domain:Fibrillar collagen NC1	6	3.70E-07	9.10E-05
SMART	EGF	17	1.20E-06	9.50E-05
INTERPRO	Peptidase M12B, ADAM-TS	7	1.60E-06	1.00E-04
INTERPRO	ADAM-TS Spacer 1	7	1.60E-06	1.00E-04
INTERPRO	Homeodomain, metazoa	11	1.80E-06	1.00E-04
UP_KEYWORDS	Wnt signaling pathway	13	1.40E-05	2.70E-04
UP_SEQ_FEATURE	propeptide:C-terminal propeptide	5	3.10E-06	6.30E-04
KEGG_PATHWAY	PI3K-Akt signaling pathway	20	1.00E-05	6.40E-04
UP_SEQ_FEATURE	compositionally biased region:Cys-rich	13	4.50E-06	7.90E-04
KEGG_PATHWAY	Protein digestion and absorption	10	2.10E-05	1.00E-03
UP_KEYWORDS	Sushi	7	6.70E-05	1.20E-03
INTERPRO	Metallopeptidase, catalytic domain	10	2.40E-05	1.30E-03
KEGG_PATHWAY	Amoebiasis	11	3.50E-05	1.40E-03
UP_SEQ_FEATURE	region of interest:Triple-helical region	6	1.10E-05	1.50E-03
INTERPRO	Laminin G domain	8	3.50E-05	1.70E-03
INTERPRO	Laminin G domain	8	3.50E-05	1.70E-03
UP_SEQ_FEATURE	domain:EGF-like 4; calcium-binding	7	1.70E-05	2.10E-03
KEGG_PATHWAY	Hypertrophic cardiomyopathy (HCM)	9	6.50E-05	2.10E-03
UP_KEYWORDS	Metal-binding	77	1.40E-04	2.30E-03
UP_SEQ_FEATURE	domain:Ig-like C2-type 3	10	2.30E-05	2.60E-03
KEGG_PATHWAY	Dilated cardiomyopathy	9	9.30E-05	2.60E-03
SMART	FN3	13	8.50E-05	2.80E-03
UP_SEQ_FEATURE	domain:EGF-like 2; calcium-binding	8	3.20E-05	3.20E-03
UP_SEQ_FEATURE	domain:TSP type-1 1	7	3.40E-05	3.20E-03
UP_SEQ_FEATURE	domain:TSP type-1 2	7	3.40E-05	3.20E-03
UP_KEYWORDS	Cleavage on pair of basic residues	13	3.70E-04	5.10E-03
KEGG_PATHWAY	Proteoglycans in cancer	13	2.10E-04	5.20E-03
UP_SEQ_FEATURE	domain:EGF-like 3; calcium-binding	7	6.30E-05	5.50E-03
INTERPRO	Concanavalin A-like lectin/glucanase, subgroup	13	1.40E-04	6.20E-03
INTERPRO	Immunoglobulin subtype 2	14	1.40E-04	6.20E-03
INTERPRO	Fibronectin, type III	13	1.40E-04	6.20E-03
INTERPRO	Leucine-rich repeat-containing N-terminal	7	1.80E-04	7.00E-03
INTERPRO	Homeobox, conserved site	12	1.80E-04	7.10E-03
GOTERM_MF_DIRECT	metalloendopeptidase activity	11	5.00E-05	7.30E-03
INTERPRO	Peptidase M12B, ADAM/reprolysin	7	2.50E-04	8.00E-03
INTERPRO	Sushi/SCR/CCP	7	2.50E-04	8.00E-03
INTERPRO	Peptidase M12B, propeptide	7	2.30E-04	8.10E-03
INTERPRO	Insulin-like growth factor-binding protein, IGFBP	5	2.40E-04	8.20E-03
INTERPRO	Homeobox protein, antennapedia type, conserved site	5	2.40E-04	8.20E-03
UP_SEQ_FEATURE	propeptide:N-terminal propeptide	4	1.00E-04	8.30E-03
UP_SEQ_FEATURE	domain:Ig-like C2-type 1	11	1.10E-04	8.40E-03
UP_KEYWORDS	Immunoglobulin domain	18	9.70E-04	1.10E-02
UP_KEYWORDS	Leucine-rich repeat	13	9.20E-04	1.10E-02
UP_KEYWORDS	Steroid metabolism	7	8.30E-04	1.10E-02
UP_SEQ_FEATURE	domain:VWFC	5	1.80E-04	1.20E-02
UP_KEYWORDS	Homeobox	13	1.10E-03	1.20E-02
INTERPRO	von Willebrand factor, type C	6	4.50E-04	1.40E-02
UP_SEQ_FEATURE	domain:IGFBP N-terminal	5	2.30E-04	1.40E-02
INTERPRO	Leucine-rich repeat, typical subtype	11	4.90E-04	1.40E-02
UP_KEYWORDS	Cholesterol metabolism	6	1.30E-03	1.40E-02
SMART	IB	5	5.60E-04	1.50E-02
SMART	TSPN	5	1.00E-03	1.60E-02
INTERPRO	Immunoglobulin I-set	10	5.70E-04	1.60E-02
SMART	LRRNT	7	7.90E-04	1.60E-02
SMART	CCP	7	8.70E-04	1.60E-02
UP_SEQ_FEATURE	domain:TSP N-terminal	5	2.90E-04	1.70E-02
SMART	VWC	6	7.40E-04	1.70E-02
UP_SEQ_FEATURE	DNA-binding region:Homeobox	12	3.40E-04	1.80E-02
UP_SEQ_FEATURE	domain:Sushi 1	6	3.30E-04	1.80E-02
INTERPRO	Insulin-like growth factor binding protein, N-terminal, Cys-rich conserved site	4	7.80E-04	2.10E-02
UP_KEYWORDS	Endoplasmic reticulum	28	2.10E-03	2.10E-02
UP_KEYWORDS	Sterol metabolism	6	2.30E-03	2.20E-02
UP_KEYWORDS	Steroid biosynthesis	5	2.40E-03	2.20E-02
GOTERM_MF_DIRECT	metallopeptidase activity	11	4.30E-04	2.40E-02
UP_SEQ_FEATURE	domain:Disintegrin	6	5.00E-04	2.50E-02
SMART	IGc2	14	1.70E-03	2.50E-02
GOTERM_MF_DIRECT	sequence-specific DNA binding	24	5.80E-04	2.50E-02
GOTERM_MF_DIRECT	calcium ion binding	26	4.20E-04	2.60E-02
UP_SEQ_FEATURE	domain:Peptidase M12B	6	5.60E-04	2.70E-02
UP_KEYWORDS	Cholesterol biosynthesis	4	3.10E-03	2.70E-02
GOTERM_MF_DIRECT	insulin-like growth factor binding	5	5.70E-04	2.80E-02
GOTERM_BP_DIRECT	cholesterol biosynthetic process	6	2.00E-04	2.90E-02
GOTERM_BP_DIRECT	negative regulation of canonical Wnt signaling pathway	9	3.80E-04	3.20E-02
GOTERM_BP_DIRECT	Wnt signaling pathway	13	3.30E-04	3.30E-02
UP_SEQ_FEATURE	short sequence motif:Antp-type hexapeptide	5	7.40E-04	3.40E-02
INTERPRO	Homeodomain	13	1.40E-03	3.40E-02
GOTERM_MF_DIRECT	platelet-derived growth factor binding	4	9.70E-04	3.80E-02
INTERPRO	Complement C1q protein	5	1.60E-03	3.80E-02
SMART	C1Q	5	2.90E-03	3.90E-02
UP_KEYWORDS	Zymogen	10	4.80E-03	3.90E-02
GOTERM_BP_DIRECT	face morphogenesis	6	5.90E-04	4.40E-02
UP_KEYWORDS	Metalloprotease	8	5.70E-03	4.50E-02
SMART	LRR_TYP	11	3.70E-03	4.50E-02

Obox jointly down-regulated following ectopic expression GO terms

Platform	Description	Count	P_Value	Benjamini
UP_KEYWORDS	DNA replication	11	1.50E-07	1.70E-05
UP_KEYWORDS	Glycoprotein	76	1.20E-06	9.00E-05
GOTERM_BP_DIRECT	DNA replication	11	4.20E-06	3.70E-03
GOTERM_BP_DIRECT	DNA replication initiation	6	1.20E-05	6.80E-03
GOTERM_CC_DIRECT	MCM complex	4	1.70E-04	1.50E-02
UP_KEYWORDS	Kinase	20	6.10E-04	1.50E-02
UP_KEYWORDS	Growth factor	8	7.00E-04	1.60E-02
UP_KEYWORDS	Nucleotide-binding	36	1.10E-03	2.00E-02
UP_KEYWORDS	ATP-binding	30	1.10E-03	2.00E-02
INTERPRO	Mini-chromosome maintenance, conserved site	4	3.70E-05	2.10E-02
UP_KEYWORDS	Cell cycle	18	1.00E-03	2.20E-02
KEGG_PATHWAY	DNA replication	6	1.50E-04	2.80E-02
UP_KEYWORDS	Tyrosine-protein kinase	7	2.00E-03	3.00E-02
SMART	MCM	4	2.40E-04	3.30E-02
INTERPRO	Mini-chromosome maintenance, DNA-dependent ATPase	4	1.50E-04	4.20E-02

J

Top 10 genes by fold change down-regulated following over-expression

	OboxA1		*OboxA4*		*OboxA7*		*CrxOS*	
	Gene	Log_2 Fold Change	Gene	Log_2 Fold Change	Gene	Log_2 Fold Change	Gene	Log_2 Fold Change
1	*Blnk*	-1.28	*Ptpn22*	-2.36	*Mmp13*	-1.94	*Dynap*	-1.54
2	*Igsf11*	-1.18	*Mmp13*	-2.14	*Ptpn22*	-1.85	*Ahrr*	-1.43
3	*Hs3st1*	-1.14	*Itga2*	-1.87	*Mmp3*	-1.57	*Krt19*	-1.26
4	*Gprc5a*	-1.13	*Dynap*	-1.64	*Hs3st1*	-1.24	*Itga3*	-1.22
5	*Clic5*	-1.12	*Fam110c*	-1.63	*Clic5*	-1.2	*Ccbe*	-1.1
6	*Itga6*	-1.12	*Areg*	-1.62	*Nptx1*	-1.17	*Aqp5*	-1.08
7	*Ngef*	-1.08	*Mmp3*	-1.53	*Fam110c*	-1.08	*Rasgrp3*	-1.02
8	*Esm1*	-1.08	*Clic5*	-1.5	*Lgr6*	-1.07	*Coch*	-1.01
9	*Sox2*	-0.99	*Serpinb2*	-1.49	*Slc14a1*	-1.06	*Nqo1*	-0.98
10	*Hmga1-rs1*	-0.99	*Upp1*	-1.42	*Areg*	-1.04	*Ngef*	-0.97

K

OboxA1		
**Gene**	**Up/Down regulated**	**Corrected P-value**
Nanog	NA	NA
Oct4/Pou5F1	NA	NA
Sox2	Down	7.23E-10
Klf4	NA	NA
Myc	NA	NA
Tead4	Down	2.64E-05
Cdx2	NA	NA
Gata3	Down	2.51E-08

OboxA4		
**Gene**	**Up/Down regulated**	**Corrected P-value**
Nanog	NA	NA
Oct4/Pou5F1	NA	NA
Sox2	Down	4.22E-13
Klf4	Down	9.47E-07
Myc	Down	1.83E-31
Tead4	Down	5.07E-06
Cdx2	NA	NA
Gata3	Down	1.31E-07

OboxA7		
**Gene**	**Up/Down regulated**	**Corrected P-value**
Nanog	NA	NA
Oct4/Pou5F1	NA	NA
Sox2	Down	2.92E-11
Klf4	NA	NA
Myc	Down	0.000000212
Tead4	Down	0.00056
Cdx2	NA	NA
Gata3	Down	0.0000216

CrxOS		
**Gene**	**Up/Down regulated**	**Corrected P-value**
Nanog	NA	NA
Oct4/Pou5F1	NA	NA
Sox2	Down	0.03
Klf4	NA	NA
Myc	NA	NA
Tead4	Down	0.006
Cdx2	NA	NA
Gata3	Down	0.006041

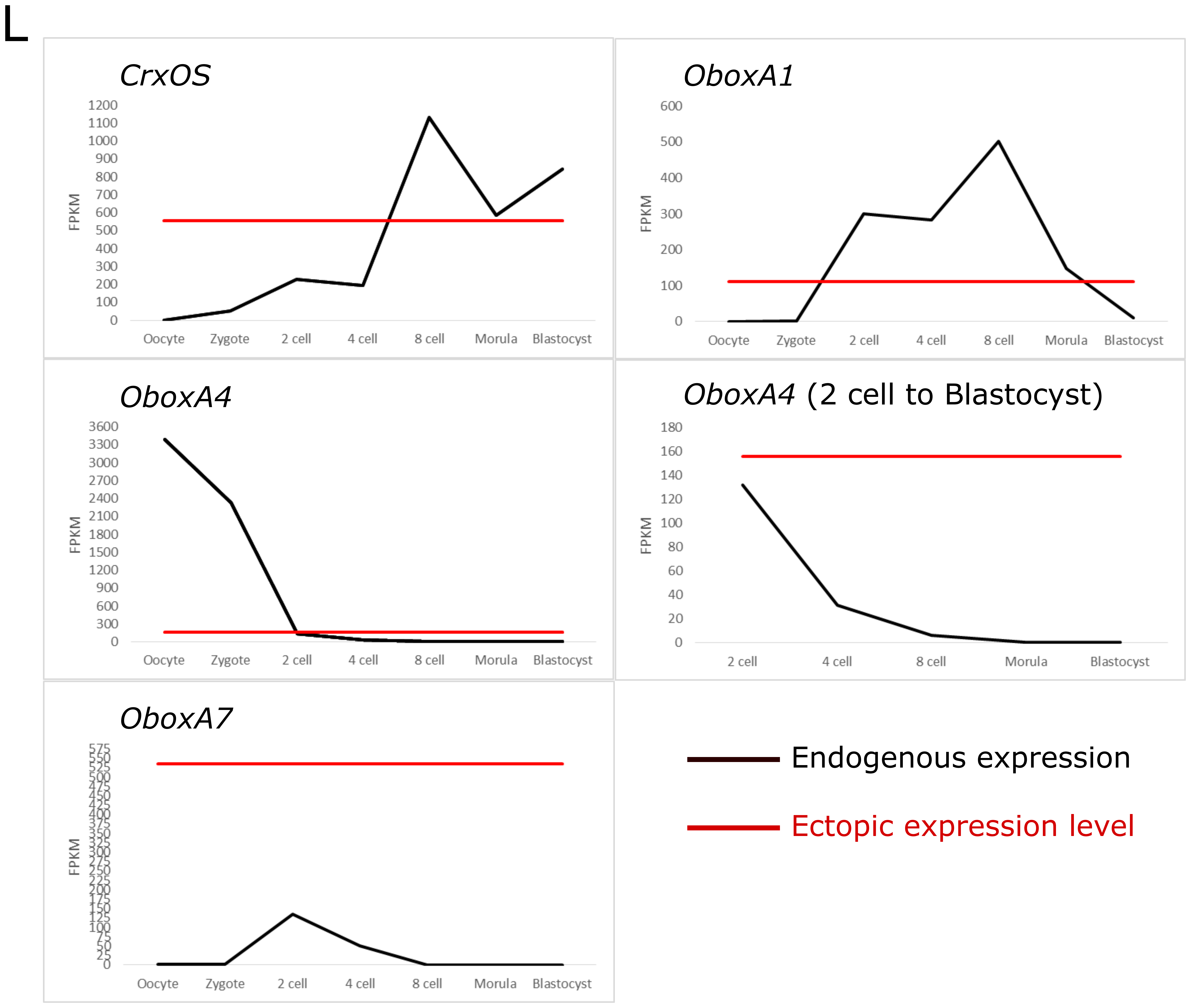
L
CrxOS
OboxA1
OboxA4
OboxA4 (2 cell to Blastocyst)
OboxA7
FPKM
Oocyte
Zygote
2 cell
4 cell
8 cell
Morula
Blastocyst
Endogenous expression
Ectopic expression level
